# Environmental assessment of mild bisulfite pretreatment of forest residues into fermentable sugars for biofuel production

**DOI:** 10.1186/s13068-016-0433-1

**Published:** 2016-01-22

**Authors:** Ikechukwu C. Nwaneshiudu, Indroneil Ganguly, Francesca Pierobon, Tait Bowers, Ivan Eastin

**Affiliations:** Department of the Environment and Forest Sciences, University of Washington, Box 351750, Seattle, WA 98195 1750 USA

**Keywords:** Pretreatment, Biomass, Biofuels, Life cycle assessment

## Abstract

**Background:**

Sugar production via pretreatment and enzymatic hydrolysis of cellulosic feedstock, in this case softwood harvest residues, is a critical step in the biochemical conversion pathway towards drop-in biofuels. Mild bisulfite (MBS) pretreatment is an emerging option for the breakdown and subsequent processing of biomass towards fermentable sugars. An environmental assessment of this process is critical to discern its future sustainability in the ever-changing biofuels landscape.

**Results:**

The subsequent cradle-to-gate assessment of a proposed sugar production facility analyzes sugar made from woody biomass using MBS pretreatment across all seven impact categories (functional unit 1 kg dry mass sugar), with a specific focus on potential global warming and eutrophication impacts. The study found that the eutrophication impact (0.000201 kg N equivalent) is less than the impacts from conventional beet and cane sugars, while the global warming impact (0.353 kg CO_2_ equivalent) falls within the range of conventional processes.

**Conclusions:**

This work discusses some of the environmental impacts of designing and operating a sugar production facility that uses MBS as a method of treating cellulosic forest residuals. The impacts of each unit process in the proposed facility are highlighted. A comparison to other sugar-making process is detailed and will inform the growing biofuels literature.

**Electronic supplementary material:**

The online version of this article (doi:10.1186/s13068-016-0433-1) contains supplementary material, which is available to authorized users.

## Background

Interest in the biochemical conversion of forest residual slash into biofuels is continually growing [1–5]. This emerging interest in using biomass for biofuel production is twofold. Firstly, healthier forests in the Pacific Northwest (PNW) require thinning and restoration work that generates wood slash [6]. Secondly, as an alternative to burning this slash into open air [7–9], this biomass can be converted into fermentable sugars towards biofuel production [3]. Biochemical conversion processes serve to remove slash piles from forests, which in turn mitigates the negative environmental impacts of decaying or burning (impacts such as long methane release and soil erosion) [10–12]. Conversion also creates drop-in alternative fuels, reducing the reliance on fossil-based fuels. A critical step in the conversion process is the release of fermentable sugars from the cellulosic biomass for downstream processing [13–15]. This is done with pretreatment techniques that degrade lignin structures in the wood allowing access to the polysaccharides for hydrolysis. Obtaining fermentable sugars is a key step in the conversion process because sugar yields significantly affect downstream fuel products [4, 15]. Additionally, sugars are a critical diverging point in many biochemical conversion pathways (also capable of being converted into chemicals like esters and carboxylic acids that are expensive to make in the petroleum industry). Understanding the environmental impacts of this sugar endpoint will be crucial for informing the growing biofuels literature.

Established pretreatment processes that liberate fermentable sugars from cellulosic biomass include dilute acid, ammonia fiber explosion, steam explosion, and hot water treatments [14, 16–18]. These conventional methods have been characterized extensively for their economic and environmental sustainability [14, 17]. However, mild bisulfite (MBS) pretreatment is an emerging technique showing similar sugar releasing efficiencies, while also offering the added advantage of utilizing well-known pulping technologies [19]. MBS pretreatment is a calcium bisulfite-based method similar to Kraft pulping or SPORL (Sulfite Pretreatment to Overcome Recalcitrance of Lignocellulose) processes where sulfurous acid is used to degrade the lignin fibers in cellulosic biomass [19]. Similarities to the conventional Kraft pulping process (low pH) gives SPORL-like processes (high pH) such as MBS an edge in development. Zhou et al. [20] report that SPORL’s advantage over other emerging techniques is its simple scale-up potential because existing infrastructure from pulping plants directly feeds into the development of the process. They also report the increased sugar yield of SPORL processes due to favorable interactions between sulfonated pulp moieties and enzymes in enzymatic hydrolysis [20]. However, these sulfite processes also create significant amounts of aqueous ligno-sulfonates, furfurals, and other organic byproducts that must be assessed for their environmental impacts [19, 20]. Although not covered in this work, these compounds can also be potentially viewed as co-products of the main product stream.

Comprehensive techno-economic assessments (TEA) and impact analysis on emerging pretreatment technologies are critical to inform feasibility of plant design and can also influence policy/venture capital interest concerning the technology [3, 21, 22]. The National Renewable Energy Laboratory (NREL) has led the way by conducting full-scale techno-economic and environmental assessments on proposed bioethanol facilities that convert corn stover into alcohol, iso-butanol, and other fuel grade compounds (using dilute acid pretreatment not MBS) [5, 22, 23]. These benchmark assessments, including plant design and optimization, were done using the Aspen Plus design software package [22]. Mass/energy flow data from this work were used in the life cycle assessment (LCA) of the plant. Within this body of work, they also detail a parallel TEA of an internal sugar production facility that would offset any fluctuations in the biofuels market (where sugar syrup could be sold as a product). Other works have also looked extensively at green-house gas (GHG) emissions and energy use of various pretreatment technologies [17, 24, 25]. However, a full environmental assessment of MBS pretreatment of woody biomass has yet to be done on a facility that produces sugar as its main product.

Environmental assessments of sugar production processes are limited in the literature. Most assessments are focused on sugars produced from sugar cane and beet sources. These typically include the land use and transportation impacts of farming and shipping the sugar. Assessments that feature lignocellulosic source materials (corn stover and wood biomass) focus on other value-added end products (ethanol, biofuels), with sugars as a starting or intermediate point within the broader process design [26]. For example, Hauschild et al. [27] analyze the impacts of turning sugar cane to bioethanol with the sugar product as a transitional step. Other studies have also assessed the use of sugars towards various kinds of biofuels and process chemicals [28]. The work by Thomas et al. [29] at the Georgia Institute of Technology shows preliminarily studies detailing a small scale production system that uses super critical water as the method of sugar production. This work focused on GHG emissions, water requirements, and energy usage of a pilot scale plant, showing comparable global warming (GW) impacts to other conventional sugar production processes. With continual improvement of sugar yields by emerging pretreatment and hydrolysis technologies, assessing the potential impacts of these sugars alone is critical for the viability of these methods as well as their potential for different markets and downstream products.

In this work, we seek to analyze the potential impacts of a prospective sugar depot, looking at the biomass pre-processing (transport/screening), pretreatment, enzyme production, enzymatic hydrolysis, separation, boiler, and water treatment of the plant that processes incoming forest residual biomass. We model a process that converts a generic blend of forest residuals using the MBS pretreatment technique. The primary focus of this assessment will be the environmental impacts of one such sugar depot located in the PNW. This will be built based on the TEA and plant design of the 2011 NREL sugar production model incorporating the MBS pretreatment technique. The assessment will use mass/energy data generated in Aspen Plus but will not include the production of enzymes for enzymatic hydrolysis (data of which will be imported from the US life cycle inventory (LCI) database).

## Process description

**Fig. 1:**
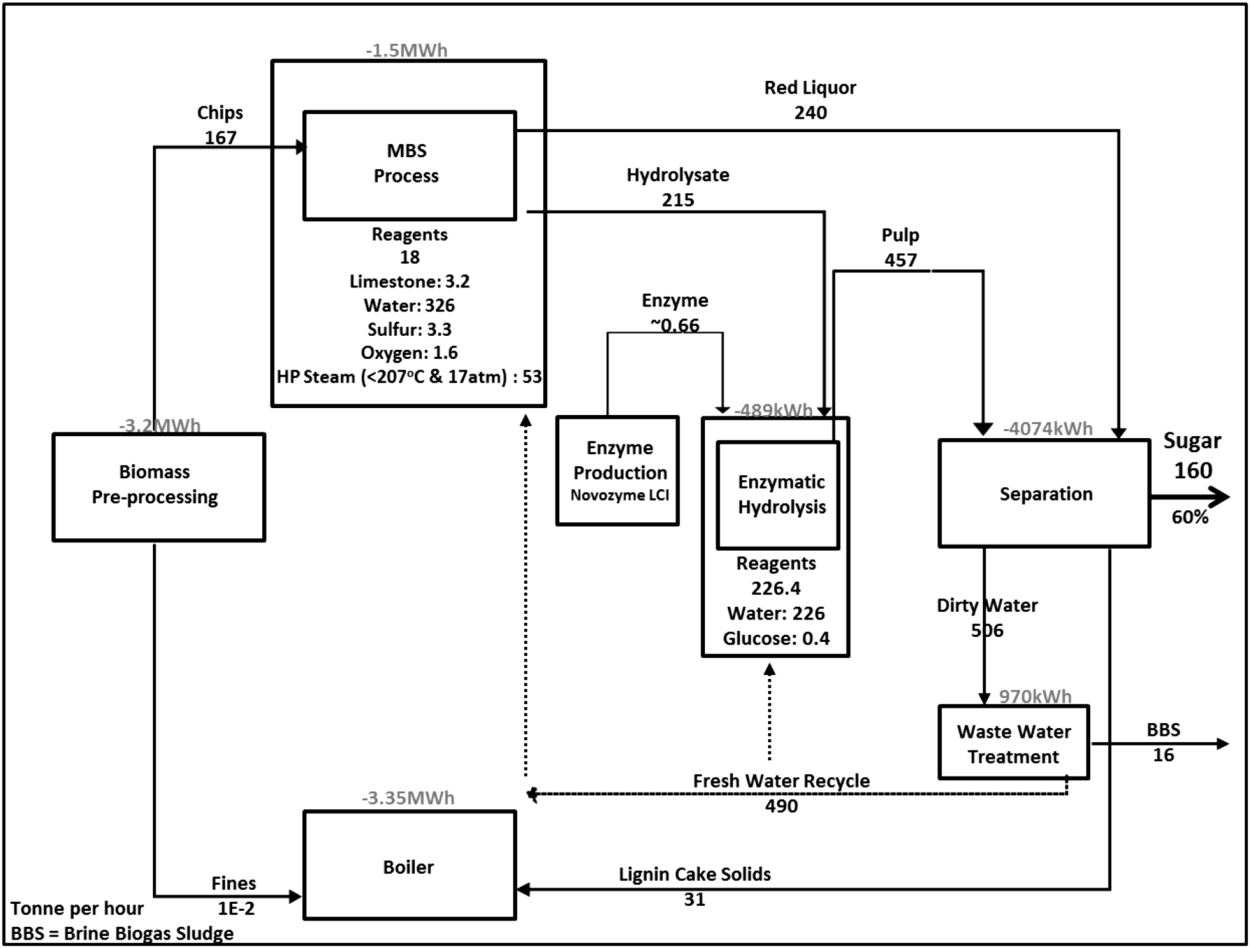
Block flow diagram of the MBS process. *Negative* signs *on power* denote usage

A detailed flow chart of the process can be seen in Fig. 1 including mass/energy flows of reagents, water, and vented vapors of all six process of the plant (transport/screening, pretreatment, enzyme production, enzymatic hydrolysis, wastewater treatment, and boiler). Based on the target biomass input of approximately 770,000 BDT (bone dry tonnes) entering the MBS conversion process, sizing and capacity adjustments were made using the automated sizing and plant mapping options available within the Aspen Plus package.

### Feedstock zone, biomass transportation, and pre-processing

The life cycle inventory (LCI) of biomass feedstock entering the processing plant is considered in this section. The analysis conducted in this section is based on the assumption that 845,000 BDT of harvested residues is fed into the biomass processing plant on an annual basis. Based on preliminary estimates (by NARA industrial consultants), it is assumed that 9 % of the biomass gets screened out (fines and rejects) of the process, and approximately 770,000 BDT of woody biomass enters the pretreatment process. The remaining 75,000 BDT of fines go to the boiler unit to be burned as hog fuel. Given the feedstock requirement, the LCI associated with the specific feedstock (FS-10—Douglas Fir/Ponderosa Pine mixture) depends on two features: (1) the feedstock zone, including the geographical region and forest types under consideration, and (2) associated feedstock logistics and in-woods processing.

#### Feedstock zone

Typical forest harvest operations in the PNW leave a considerable volume of unused woody biomass in the forest in the form of treetops and branches. These harvest residues are generally collected into slash piles and treated as part of a regional forest fire mitigation mandate. The activities of burning the non-merchantable material are designed to prevent the greater release of emissions through wildfire (Oneil and Lippke 2010) which would occur if large amounts of residuals are left in forest. The predominant method for most private land managers is to pile and burn the material. On national forest lands, piling and burning are used on gentle slopes and broadcast burning (burning of scattered slash) on steep slopes (Oneil and Lippke 2010). Slash pile burning releases the carbon sequestered in the woody biomass into the air in form of carbon dioxide (CO_2_) which has an impact on GW. Moreover, conducting broadcast burns is labor intensive, time consuming and substantially increases forest management costs.

Removing the harvest residuals from forest greatly reduces the need for slash pile burning which would potentially reduce particulate/smoke emissions. Geographical location, regional vegetation, and topographical characteristics significantly affect the environmental impacts associated with collecting and transporting the woody residues from the forest landing (slash piles) to the biomass processing facility. The woody biomass feedstock zone used in this study includes the eastern Washington, northern Idaho, and western Montana region (hereafter referred to as the “inland west region”).

#### Feedstock logistics

Biomass transportation and in-woods processing/handling of the woody residual biomass can have an influence on the overall environmental performance of the feedstock. Based on forest management practices, topography, and existing road networks in the inland west region, a series of biomass transportation scenarios are considered. Emissions generated as well as energy used was calculated for each of the feedstock handling and transportation scenarios to identify optimal solutions that minimized environmental burdens. A benchmark scenario, based on the most likely situation in the region, is presented in this paper [30]. The harvest system and in-woods feedstock handling benchmark scenario are presented in Ganguly et al.

The chosen benchmark scenario indicates that the loose residues are transported from the primary landing to the secondary landing in a 30-cubic yard (CY) dump truck, where they are chipped using a large chipper. Residuals must be transported from a primary to secondary landing (where the chipper and direct loader are located) because the 120 CY chip vans cannot navigate the forest spur road. The chipped residues are directly loaded into a 120 CY chip van and transported to the pretreatment facility. Given the target annual feedstock requirement (845,000 BDT residuals) and the estimated availability of harvest residues in the region, an average distance of 75 miles is estimated from the primary landing to the processing facility [30]. The distribution of road types, respective distances, and vehicle speeds are presented in Ganguly et al. [30]. It should be noted that although the feedstock LCA is reported in bone dry units, a 35 % feedstock moisture content is assumed when calculating truck capacity and associated fuel consumption. Finally, the feedstock handling at the pretreatment facility includes getting bales of chipped woody biomass onto an electrically powered conveyor belt, on which the wood is screened and fed into the plant.

### Mild bisulfite pretreatment

After feed handling and screening, the wood chips are fed into a batch pretreatment reactor that uses electricity, high-pressure steam (above 207 °C and 17 atm), and reagents (calcium bisulfite, sulfurous acid, water, and oxygen). The calcium bisulfite and sulfurous acids are created onsite by two stage reactions. Firstly, sulfur (S) powder is burned at ~1400 °C producing sulfur dioxide (SO_2_). The heat generated from this reaction will be used to create steam that heats the biomass digestion reaction. Secondly, limestone [Ca (CO)_3_] is then reacted with the sulfur dioxide to produce calcium bisulfite, sulfurous acid, and CO_2_. These two reagents along with water and oxygen are reacted with the biomass in a digester for 4–14 h at a pH of ~2 before being sent by conveyor to be washed. After the wash, the supernatant is collected as spent sulfite liquor (SSL), while the wet pulp is flashed and sent into enzymatic hydrolysis tanks.

### Enzyme production

The process model in the 2011 NREL report accounted for the production of the Cellulase enzyme. As stated in the report, this scheme entailed using a T. reesei-like fungus to create the Cellulase enzyme. An aerobic fermentation is model using a feedstock of glucose and fresh water [22]. Media and a small amount of purchased Cellulase are used to induce Cellulase production. Created Cellulase enzymes are then sent into enzymatic hydrolysis tanks. However, for this environmental assessment, data for the Novozyme Cellulase enzyme production process was used directly from the US-LCI database. Enzymes from Novozymes (the Denmark-based enzyme production company) are made in house using a production scheme similar to that model in the NREL report.

### Enzymatic hydrolysis

Enzymes and reagents (water, lime) are added to eight enzymatic hydrolysis batch reactors where they are left to sit for 72 h (lime for pH adjustment). Enzyme input into the unit is approximately 0.66 TPH (tonne per hour). Reagents, water, and lime are added to each batch reactor and are typically stirred for increased sugar yields (~80 % conversion rate).

### Separation

Lignin solids in the sugar slurry are separated out by centrifuge, and the remaining liquid is then recombined with the washed ligno-sulfonate “red liquor” stream. The resulting output stream is then concentrated with a three-stage triple effect evaporator operating at three pressure stages. This creates our concentrated 60 % sugar product stream with 40 % being water and other aqueous contaminants, 1 kg of which serves as the functional unit for the assessment. The gas evaporate waste is condensed back into a liquid and sent to the wastewater treatment facility.

### Wastewater treatment

Wastewater treatment is adapted from the closed-loop system used in the modified NREL model [23], using proportions of the input and output streams as well as the electricity usage of the unit. This treatment process uses digestion, anaerobic/aerobic treatment, as well as filtration. The process is assumed to recycle all of the process water with no discharge of waste water outside the system boundaries. Waste streams of biogas go into the boiler, while the brine waste is discharged.

### Boiler

The boiler process is adapted from the NREL process design consisting of a stoker fired boiler that burns biogas, sludge (from water treatment), fines (screening), and lignin cake (from separation) towards generation of electricity that can be fed back to the energy grid. Burning of these components generates steam that is used in a steam turbine. For this process, reasonable estimates were used for its efficiency and outputs based on the NREL process design. Within the assumptions made in Table 1, proportions were taken from the inputs and outputs of the NREL design and used to estimate outputs for the MBS process (the significant assumption being that the inputs from the MBS process are similar to that of dilute acid). With this assumption, the only deviation from the NREL boiler would be the input lignin content.

**Table 1:** Assumptions made for the development of the sugar production model

Process	Assumptions
Biomass generation	845,000 tonnes/year to plant, with 75TPY fines screened to boiler. 770,000 going to the bioconversion process
Boiler	Efficiency and yields are comparable to the 2011 NREL Aspen model
MBS pretreatment	Biomass is at 50 % water content. FS-10, a blend of softwoods (Douglas Fir, Ponderosa Pine). Byproducts from the reaction will be comparable to those from NREL’s dilute acid model
Wastewater treatment	Efficiency and yields are comparable to the 2011 NREL Aspen model

## Results and discussion

Using the data generated in the Aspen Plus (data for biomass pre-processing, pretreatment, hydrolysis, and separation units) and data from the US-LCI for enzyme production, as well as assumptions based on the NREL wastewater treatment and boiler units, the impacts of the proposed sugar production facility across all seven impact categories are analyzed. The Aspen Plus data were integrated into SimaPro by simply adjusting all input and output streams to the functional unit of 1 kg of sugar product. Table 2 shows impacts across categories: GW, eutrophication, acidification, smog formation, ozone depletion, carcinogens, non-carcinogens, respiratory effects, and ecotoxicity. We focus on the eutrophication and GW impacts because of their significance to the proposed sugar plant and the assumptions made for the model (CO_2_ and water discharge). GW impacts are tied to the significant amount of carbon dioxide produced in the process, and eutrophication is strongly influenced by the large amount of contaminated water which could be released by the system. The potential health and air quality impacts for three prominent but ancillary categories (smog, acidification, and ecotoxicity) will also be addressed later in the article.

**Table 2:** Nine impact categories assessed for the sugar production facility

Impact category	Unit	Total
Ozone depletion	kg CFC-11 eq	3.73E-08
Global warming	kg CO_2_ eq	0.353098
Smog	kg O_3_ eq	0.058057
Acidification	mol H + eq	0.118889
Eutrophication	kg N eq	0.000169
Carcinogens	CTUh	3.38E-09
Non-carcinogens	CTUh	2.22E-08
Respiratory effects	kg PM10 eq	0.000238
Ecotoxicity	CTUe	0.427871

### Global warming

**Fig. 2:**
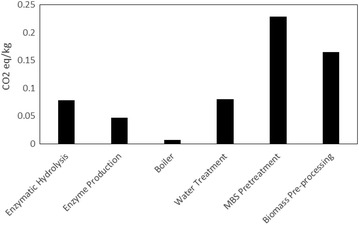
Process contribution to global warming. Six main units of the sugar process are shown with their corresponding GW impacts (measured in CO_2_ equivalents/kg)

A comparison of global warming potential (GWP) for our model system is shown in Fig. 2. The bar graph in the figure is calculated in kilogram carbon dioxide (kg CO_2 eq_) equivalents and normalized to the sugar product output. The figure shows the calculated GWP of the major processes in sugar production including biomass pre-processing, MBS pretreatment, enzyme production, enzymatic hydrolysis, wastewater treatment, and boiler. We see from the figure that the greatest contributors to the GWP of the proposed facility are biomass pre-processing and MBS pretreatment. The biomass pre-processing GWP is mainly influenced by the diesel-range fuels used to prepare and transport the forest residuals before arriving at gate. These may change depending on the possible logistical options for forestry operations and transportation scenarios available. The MBS pretreatment process, which has the highest GW impact, is predominantly influenced by the high-pressure steam used for the process. These impacts are shown in more detail [see Additional file 1]. The reagents used such as limestone, water, acid were only marginally influential to the observed GWP when compared to the impact of the high-pressure steam needed for the process. Lastly, it is worth noting that although this steam is modeled as an input from the technosphere, the actual process does generate most of its own high-pressure steam from the modeled sulfur boiler. However, it was modeled as such to show the absolute impact of MBS requirements within the plant. This could be adjusted to show the displacement credit possibly gained from displacing the steam requirements.

Figure 2 also shows the less impactful unit processes to GW (boiler, enzymatic hydrolysis, enzyme production, and water treatment). Modeling the biogas and dirty water as inert (because they feed back into the system) means that most of the GW impact of the process is coming from the electricity usage. The enzyme production and boiler (burning hog fuel and lignin cake) units have the least GHG emissions. The LCI for Cellulase from the US-LCI database only shows a marginal release of carbon dioxide into the environment. A majority of the boiler’s impact is primarily due to the electricity used by the turbo generator. Assumptions made about the composition of the lignin cake could be very influential to this result and will be addressed later in the document. Lastly, impacts from enzymatic hydrolysis can be attributed to the medium pressure steam and other reagents (quicklime, water, etc.) required for the process.

#### Sugar product

The overall GW impact of the sugar product leaving the production facility was assessed. The resultant stream does not account for any of the burdens from the aqueous contaminants (sulfonated lignin, furfural, acetic acid) that remain in the concentrated sugar product stream. The sugar stream along with the aqueous contaminants is to be supplied to an external alcohol production facility, as is proposed in the NREL corn stover model. The value of the GWP of 1 kg sugar product is calculated to be 0.353. This number includes the displacement credit taken from the grid-bound electricity being produced by the boiler. We assess the reasonableness of this value by comparing it to other values reported in the literature for similar technologies. Although very limited, some values were obtained for the specified system (biomass to sugars only). Thomas et al. [29] yield a GWP value of 0.522 for its super critical sugar production process. Tao et al. [17] show a broader range of GWP between 0.9 and 2.5 kg CO_2 eq_/kg sugar for a wide gamut of pretreatment technologies (NREL 2011 SOT, AFEX, dilute acid, etc.). We see that the MBS and super critical water values are lower than those from these other sugar production processes. Accounting for the added displacement credit from the sold electricity, we see that we get a value of 0.518 for our MBS process, which is similar to the super critical process. A number of factors including impact assessment methods and modeling assumptions could explain these differences in resulting GWP.

### Eutrophication

**Fig. 3:**
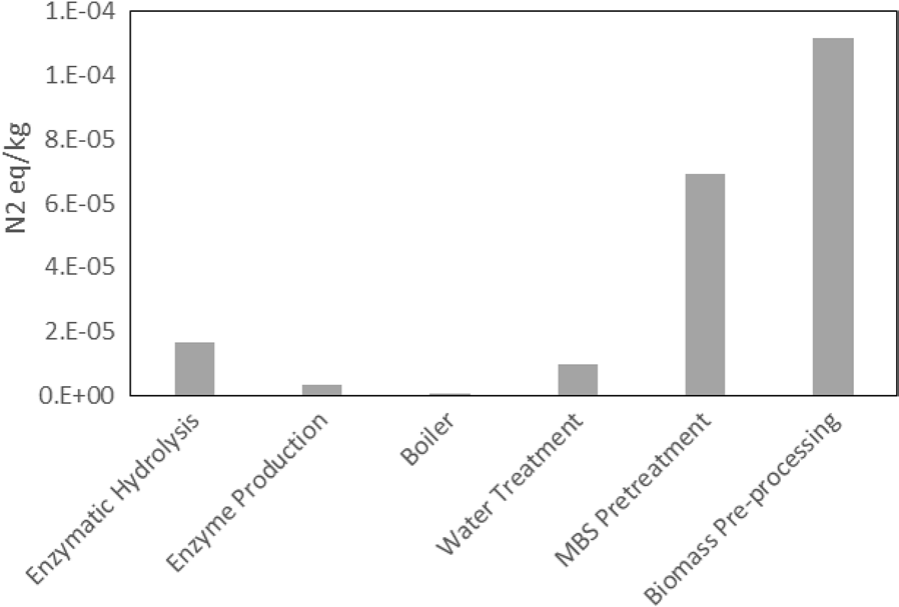
Process contribution to eutrophication. Six main units of the sugar process are shown with their corresponding eutrophication impacts (measured in Nitrogen equivalents/kg)

Assessing eutrophication impacts on local and global bodies of water is critical for the proposed sugar production facility. We see that the large volume of water being used in the process would be most influential within this category. This work focuses on a proposed nth plant facility, so we assume a stock of well water to start the process. As shown in Fig. 1, we assume that most of the water is being treated, recovered, and recycled back into the system at a rate of 490 tonnes per hour, meeting a significant portion of the process’s water requirements. Recycled water is redistributed back into the system based on individual water requirements of each unit process. Figure 3 shows the six major processes and their eutrophication impacts, calculated in terms of gram per unit nitrogen (N) equivalents (TRACI—Tool for the Reduction and Assessment of Chemical and Other Environmental Impacts ). We see from the figure that biomass pre-processing and MBS pretreatment are processes that contribute the most to impacts in the eutrophication category. The high eutrophication impact of the biomass pre-processing unit is caused by the associated burdens of the diesel-range fuels used by the machinery and trucks that process/transport the biomass. The same reasoning can also be used to describe the MBS pretreatment process impact; outside of the start-up water requirements that are simply recycled and re-used within the system, the process also uses water to wash the sulfur treated biomass and within heat exchangers for cooling. These all do contribute to the observed impacts because water soluble sulfur-based moieties as well as other chemicals (acetic acid, ammonia, and furfurals) are present in these aqueous streams, which could have significant impacts if released into local bodies of water.

Outside of the biomass pre-processing and MBS pretreatment, the other processes (wastewater treatment, enzymatic hydrolysis, boiler, and enzyme production) seem to have relatively low eutrophication impact in the sugar production process. Of the four listed, enzymatic hydrolysis and waste water treatment have the next greatest impact on eutrophication. This could be due to the water used to wash the sulfonated pulp and the possibility that “dirty water” (containing ammonia and nitrogen-based compounds) to be discharged into local streams and rivers. We also see that the impact of the wastewater treatment unit is marginal compared to the MBS and hydrolysis processes, even though a significant amount of water is being processes by the unit. This is because our assumed wastewater treatment process emits only brine (table salt) to the environment, unlike the boiler that emits ash and other nitrogen containing moieties. Additionally, the low impact of the water treatment facility is highly dependent on the assumption we made about the efficiency of the unit (our treatment unit being relatively similar to the NREL wastewater treatment process). In conventional pulping processes, the spent sulfite liquor (SSL) from the pretreatment process is not sent to be treated. Most processes find ways to use this stream. In the Kraft process, the “black or brown liquor” is treated with various chemicals to create more benign solids or are just disposed of in appropriate means [31, 32]. For more acidity processes like MBS, a “red liquor” is created also primarily composed of sulfonated lignin compounds. At the right concentration, the SSL can be sold as a cement additive [33]. This warrants further investigation as this stream accounts for a significant volume percentage of the “dirty water” begin treatment in the process. The uses of spent sulfite liquor streams are still emerging and will need to be fully explored to determine its place in the growing biofuels and co-products literature.

#### Sugar product

The overall impact of the sugar product leaving the production facility was assessed within the eutrophication category. A normalized numerical value of 0.000201 kg N was obtained for the process. Due to a lack of eutrophication data from pretreatment technologies, these values were compared to beet and sugarcane products. We see that the process modeled here is an order of magnitude lower than cane (0.00109 kg N) and beet (0.00488 kg N) processes. The difference may be due to a higher proportion of water and fertilizer used in sugar cane and beet production. Assumptions based on the NREL dilute acid model may not necessarily transfer to the MBS process, especially in terms of water usage. Although the value of the impact category seems negligible as compared to some of the other categories in Table 1, it is important to note that this value is skewed based on the assumptions made about the treatment of the sulfonated aqueous stream coming from the pretreatment process. This would greatly affect the value of the number as the volume of the “red liquor” stream is significant. This can be sold as a cement additive. However, it is important to note that, as shown in Fig. 3, the overall eutrophication impacts of all these processes are relatively small as compared to beet and sugar cane processes. This may illustrate the need for efficient use of land and water resources in these types of proposed sugar production facilities.

### Health and air impacts

Although not directly related to the bioconversion processes, three impact categories (smog, acidification, and ecotoxicity) in Table 1 do show significant impacts in the MBS sugar-making process. These impacts are shown to primarily come from the pretreatment and biomass pre-processing steps (processing and transportation of the woody biomass in the forest) with very slight impacts associated with fuels used in the processes. We also notice that for categories of ozone depletion, carcinogens, non-carcinogens, and respiratory effects, pre-processing processes and MBS pretreatment units carry a major part of the burdens (this does not include aspects that are unique to the conversion process). However, it is worth noting that under various conditions within the plant (for example, if evaporate streams were vented rather than condensed and sent to the wastewater treatment facility), these impact categories would be more integral to this kind of assessment.

## Conclusions

A “cradle to gate” life cycle analysis was conducted for sugar production from forest residue slash using MBS pretreatment. We analyze the process for its contributions to all impact categories within the TRACI assessment method, with specific focus on GW and eutrophication. Within the process, we found that the most impactful processes in both impact categories are the biomass pre-processing before it enters the plant site and the MBS pretreatment. This not only highlights the significant impact of more efficient transportation, but it also shows the environmental impacts of grinders, chippers, and other harvesting machinery that run on diesel-range fuels. Within MBS pretreatment, we see that a majority of the impacts are attributed to the high-pressure steam being used in the process. Although the steam requirements could be met by the steam outputs of the sulfur and biomass boilers, we show here the uncredited impacts of MBS conversion relative to other processes as a worst case scenario as well as to detail the overall needs of the MBS pretreatment. We also see that the sugar-making process carries burdens (within both categories) from reagents like water, steam and chemicals. Although the assumptions made in this work create a convenient closed-loop system to work with, we acknowledge that the large amount of water (which could potentially contain nitrogen-based compounds from the sugar production process) needed for the process may have a more significant impact in the eutrophication category. During the presented life cycle assessment of forest residual MBS sugar, we show that the GW impact (0.353 and 518 kg CO_2 eq_) is lower than other pretreatment methods. We attributed this difference to the assumptions made about water treatment and lignin utilization. We also show that the impacts on eutrophication were significantly low when compared to beet and cane sugars. More importantly, due to the limited data on sugars products from cellulosic sources, this work highlights the possible impacts of a proposed sugar depot. Being assessed as a product, not just an intermediate, leaves much needed flexibility for emerging technologies within the ever-changing biofuels landscape. This work not only shows the importance of assessing the impacts of biomass to sugar production processes, but it also highlights the importance of water utilization and discharged. The strength of MBS and other acidic based process will be strongly tied to the ability to utilize SSL streams from such systems, which seems very likely with MBS pretreatment.

## Methods

### Goal and scope

The goal of this study was to develop a life cycle impact assessment of softwood-based sugar syrup produced at 60 % concentration. We looked to examine environmental impacts that would result from the MBS sugar production process on a cradle-to-gate basis. More specifically, we sought to assess the impacts of seven distinct steps of the sugar production process: (1) biomass pre-processing, (2) pretreatment, (3) enzyme production, (4) enzymatic hydrolysis, (5) separation, (6) wastewater treatment, and (7) boiler energy production. The environmental impacts calculated include GW, GHG emissions (kilograms of CO_2_ equivalents), and eutrophication (N equivalents). The functional unit used in this analysis is 1 kg of sugar product from forest residuals. The pretreatment, hydrolysis, and separation processes were modeled in Aspen Plus at an industrial scale (annually, 770 green tonnes of woody biomass converted to sugar syrup). Data for biomass pre-processing, enzyme production, wastewater treatment, and the boiler were generated outside the Aspen model (US-LCI database, NREL report). The TRACI impact assessment method was used within the SimaPro 8 platform to quantify the life cycle environmental impacts.

### System boundary

**Fig. 4:**
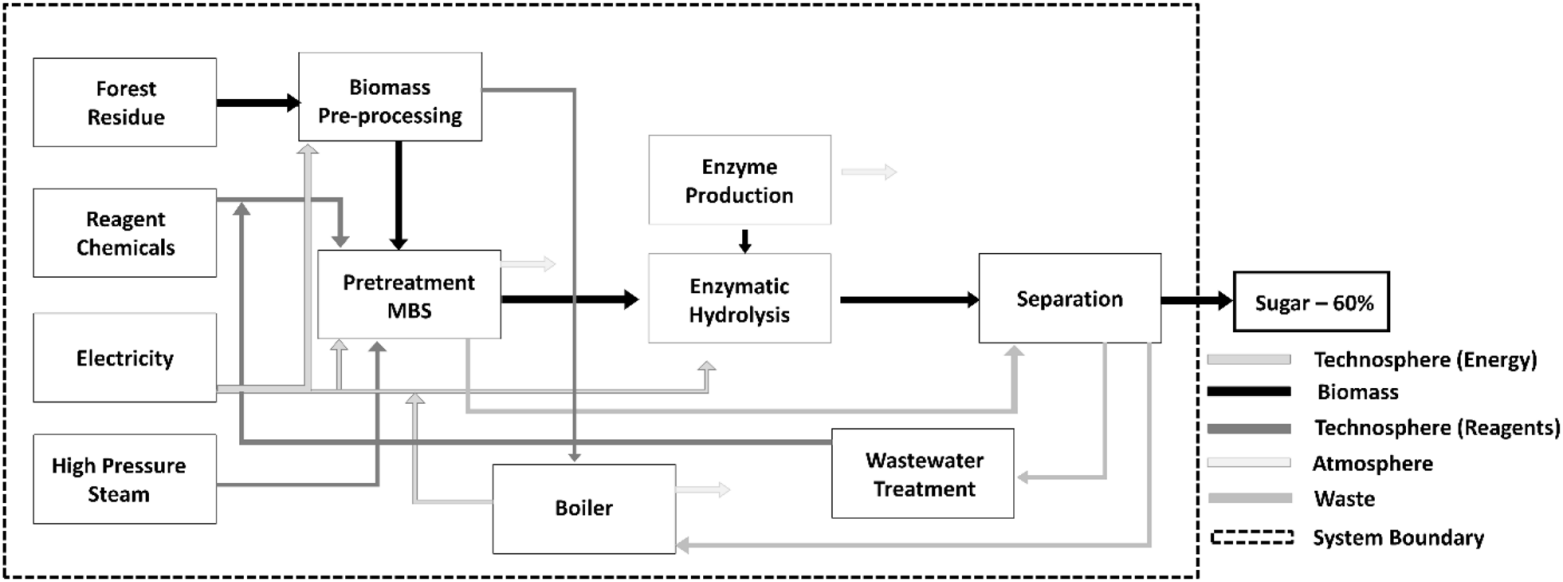
System flow sheet and boundaries

To quantify GHG emissions from the production of sugar, a cradle-to-gate system boundary was adopted. In this work, we define “cradle” as the source of waste/residual woody biomass (slash piles) at the forest landing, where only the burdens of the operations to grind and transport the slash pile are included. The residual contaminants left in the concentrated sugar product are outside the scope of this study. The amount of solids sent to the boiler to generate energy is estimated using specified metrics from the NREL 2011 model. The proposed geographic region for the study will be the PNW. The cut-off rule used is 95 % on a mass basis. The duration of the study is over a one-year period. A complete diagram of the processes covered in this study is detailed in Fig. 4. The dashed lined box signifies the processes within the system boundary of the assessment.

### Impact assessment method

Simulated process models in Aspen Plus were used to generate inventory results for the MBS conversion process assessed in the study. Using data generated in the process model, SimaPro 8 was used to calculate the environmental impacts using the US Life Cycle Inventory (US-LCI) database. The TRACI impact assessment method was used because it is an US-specific, EPA-recognized impact assessment method [28]. SimaPro software accesses the US-LCI database from the National Renewable Energy Laboratory and internally uses the TRACI method to quantify impacts.

### Inventory analysis

The data for mild bisulfite pretreatment were obtained from a fully developed process model in Aspen Plus. The boiler and wastewater treatment units were not modeled in Aspen, but energy outputs for burning lignin and water treating efficiencies were estimated from the NREL 2011 report and are included in the assessment. The collected data for the different processes, such as mass flows, reaction yields, and utilities (steam, electricity, etc.), were modeled and estimated in Aspen Plus. Although the Aspen simulations were modeled in green tonnage, the subsequent assessment is evaluated in the conventional bone dry tonnage (BDT) of the system.

### Assumptions

Assumptions made for various aspects of the model allow for a reasonable estimate of overall material flow. Table 1 details the assumptions used to simplify the model. We assumed an 845,000 green metric tonnes per year (TPY) biomass feed rate with 770,000 TPY going into the process and 75,000 TPY discarded and used as hog fuel (based on grant specifications for the proposed facility). We also assumed that inputs/outputs to the boiler and water treatment units (dirty water, lignin cake, reagents, and air) were relatively similar to those in the NREL model. To the first order, this assumption is reasonable because the yields of sugars between different feedstocks are relatively similar, and the most significant difference between NREL corn stover feedstock and the FS-10 feedstock used in this study is lignin content. Therefore, adjustments were made to account for the greater lignin content in FS-10 feedstock, which translates to a change in lignin cake and ligno-sulfonate output. For the wastewater treatment process, we also assumed based on the NREL model that the composition of “dirty water” going into the unit produces the same ratio and consistency of clean water, sludge, brine, and biogas.

## Additional file

10.1186/s13068-016-0433-1 Reagent impacts. This is additional data from the study showing some of the eutrophication and global warming impacts of Reagents used in the study (i.e. limestone, medium/high pressure steam, quicklime, water).

## Abbreviations

MBS: mild bisulfite
PNW: Pacific Northwest
SPORL: Sulfite Pretreatment to Overcome Recalcitrance of Lignocellulose
TEA: techno-economic assessments
NREL: National Renewable Energy Laboratory
LCA: life cycle assessment
GHG: green-house gas
LCI: life cycle inventory
BDT: bone dry tonne
NARA: Northwest advanced renewable alliance
CY: cubic yard
SSL: spent sulfite liquor
TPH: tonne per hour
GWP: global warming potential
AFEX: ammonia fiber explosion
SOT: state of technology
TRACI: Tool for the Reduction and Assessment of Chemical and Other Environmental Impacts

## References

[1] Dutta K, Daverey A, Lin JG (2014). Evolution retrospective for alternative fuels: first to fourth generation. Renew Energy.

[2] Lappas AA, Iatridis DK, Vasalos IA (2011). Production of liquid biofuels in a fluid catalytic cracking pilot-plant unit using waxes produced from a biomass-to-liquid (BTL) process. Ind Eng Chem Res.

[3] Sunde K, Brekke A, Solberg B (2011). Environmental impacts and costs of woody biomass-to-liquid (BTL) production and use—a review. For Policy Econ.

[4] Mood SH, Golfeshan AH, Tabatabaei M, Jouzani GS, Najafi GH, Gholami M, Ardjmand M (2013). Lignocellulosic biomass to bioethanol, a comprehensive review with a focus on pretreatment. Renew Sustain Energy Rev.

[5] Tao L, Aden A, Humbird D. Economics of current and future biofuels. Abstracts Pap Am Chemical Soc. 2009;238.

[6] Zamora-Cristales R, Sessions J, Boston K, Murphy G (2015). Economic optimization of forest biomass processing and transport in the Pacific Northwest USA. For Sci.

[7] Busse MD, Shestak CJ, Hubbert KR (2013). Soil heating during burning of forest slash piles and wood piles. Int J Wildland Fire.

[8] Creech MN, Kirkman LK, Morris LA (2012). Alteration and recovery of slash pile burn sites in the restoration of a fire-maintained ecosystem. Restor Ecol.

[9] Johnson BG, Johnson DW, Miller WW, Carroll-Moore EM, Board DI (2011). The effects of slash pile burning on soil and water macronutrients. Soil Sci.

[10] Massman WJ. Modeling soil heating and moisture transport under extreme conditions: forest fires and slash pile burns. Water Resources Res. 2012; 48.

[11] Jaafar Z, Loh TL (2014). Linking land, air and sea: potential impacts of biomass burning and the resultant haze on marine ecosystems of Southeast Asia. Glob Change Biol.

[12] Preston CM, Smernik RJ, Powers RF, McColl JG, McBeath TM (2011). The decomposition of windrowed, chipped logging slash and tree seedling response: a plant growth and nuclear magnetic resonance spectroscopy study. Org Geochem.

[13] Agbor VB, Cicek N, Sparling R, Berlin A, Levin DB (2011). Biomass pretreatment: fundamentals toward application. Biotechnol Adv.

[14] Eggeman T, Elander RT (2005). Process and economic analysis of pretreatment technologies. Bioresour Technol.

[15] Lloyd TA, Wyman CE (2005). Combined sugar yields for dilute sulfuric acid pretreatment of corn stover followed by enzymatic hydrolysis of the remaining solids. Bioresour Technol.

[16] Gao DH, Chundawat SPS, Uppugundla N, Balan V, Dale BE (2011). Binding characteristics of Trichoderma reesei Cellulases on untreated, ammonia fiber expansion (AFEX), and dilute-acid pretreated lignocellulosic biomass. Biotechnol Bioeng.

[17] Tao L, Aden A, Elander RT, Pallapolu VR, Lee YY, Garlock RJ, Balan V, Dale BE, Kim Y, Mosier NS (2011). Process and technoeconomic analysis of leading pretreatment technologies for lignocellulosic ethanol production using switchgrass. Bioresour Technol.

[18] Nwaneshiudu IC, Schwartz DT (2015). Rational design of polymer-based absorbents: application to the fermentation inhibitor furfural. Biotechnol Biofuels.

[19] Gao J, Anderson D, Levie B (2013). Saccharification of recalcitrant biomass and integration options for lignocellulosic sugars from Catchlight Energy’s sugar process (CLE Sugar). Biotechnol Biofuels.

[20] Zhou HF, Zhu JY, Luo XL, Leu SY, Wu XL, Gleisner R, Dien BS, Hector RE, Yang DJ, Qiu XQ (2013). Bioconversion of beetle-killed lodgepole pine using SPORL: process scale-up design, lignin coproduct, and high solids fermentation without detoxification. Ind Eng Chem Res.

[21] Mielenz JR (1997). Feasibility studies for biomass-to-ethanol production facilities in Florida and Hawaii. Renewable Energy.

[22] Nguyen QA, Dickow JH, Duff BW, Farmer JD, Glassner DA, Ibsen KN, Ruth MF, Schell DJ, Thompson IB, Tucker MP (1996). NREL/DOE ethanol pilot-plant: current status and capabilities. Bioresour Technol.

[23] Humbird D, Mohagheghi A, Dowe N, Schell DJ (2010). Economic impact of total solids loading on enzymatic hydrolysis of dilute acid pretreated corn stover. Biotechnol Prog.

[24] McKechnie J, Pourbafrani M, Saville BA, MacLean HL (2015). Exploring impacts of process technology development and regional factors on life cycle greenhouse gas emissions of corn stover ethanol. Renew Energy.

[25] Pourbafrani M, McKechnie J, Shen T, Saville BA, MacLean HL (2014). Impacts of pre-treatment technologies and co-products on greenhouse gas emissions and energy use of lignocellulosic ethanol production. J Clean Prod.

[26] Binder JB, Raines RT (2010). Fermentable sugars by chemical hydrolysis of biomass. Proc Natl Acad Sci USA.

[27] Ometto AR, Hauschild MZ, Lopes Roma WN (2009). Lifecycle assessment of fuel ethanol from sugarcane in Brazil. Int J Life Cycle Assess.

[28] Gopal AR, Kammen DM (2009). Molasses for ethanol: the economic and environmental impacts of a new pathway for the lifecycle greenhouse gas analysis of sugarcane ethanol. Environ Res Lett.

[29] Thomas VM, Realff MJ, Choi DG, Luo D: A supercritical water approach to cellulosic sugars: lifecycle energy, greenhouse gas and water implications. In: School of Industrial and Systems Engineering. Georgia Institute of Technology. 2012;10955.

[30] Pierobon F, Ganguly I, Anfodillo T, Eastin IL (2014). Evaluation of environmental impacts of harvest residue-based bioenergy using radiative forcing analysis. Forestry Chron.

[31] Jonsson AS, Nordin AK, Wallberg O (2008). Concentration and purification of lignin in hardwood kraft pulping liquor by ultrafiltration and nanofiltration. Chem Eng Res Des.

[32] Wallberg O, Jonsson AS, Wimmerstedt R (2003). Ultrafiltration of kraft black liquor with a ceramic membrane. Desalination.

[33] Konduri MKR, Fatehi P (2015). Production of water-soluble hardwood Kraft Lignin via Sulfomethylation using formaldehyde and sodium sulfite. ACS Sustainable Chem Eng.

