# Supplementary figures and images for: Environmental assessment of mild bisulfite pretreatment of forest residues into fermentable sugars for biofuel production
Source: Biotechnol Biofuels. 2016 Jan 22;9:15. doi: 10.1186/s13068-016-0433-1 (PMC4722614; doi:10.1186/s13068-016-0433-1)

## Slide 1
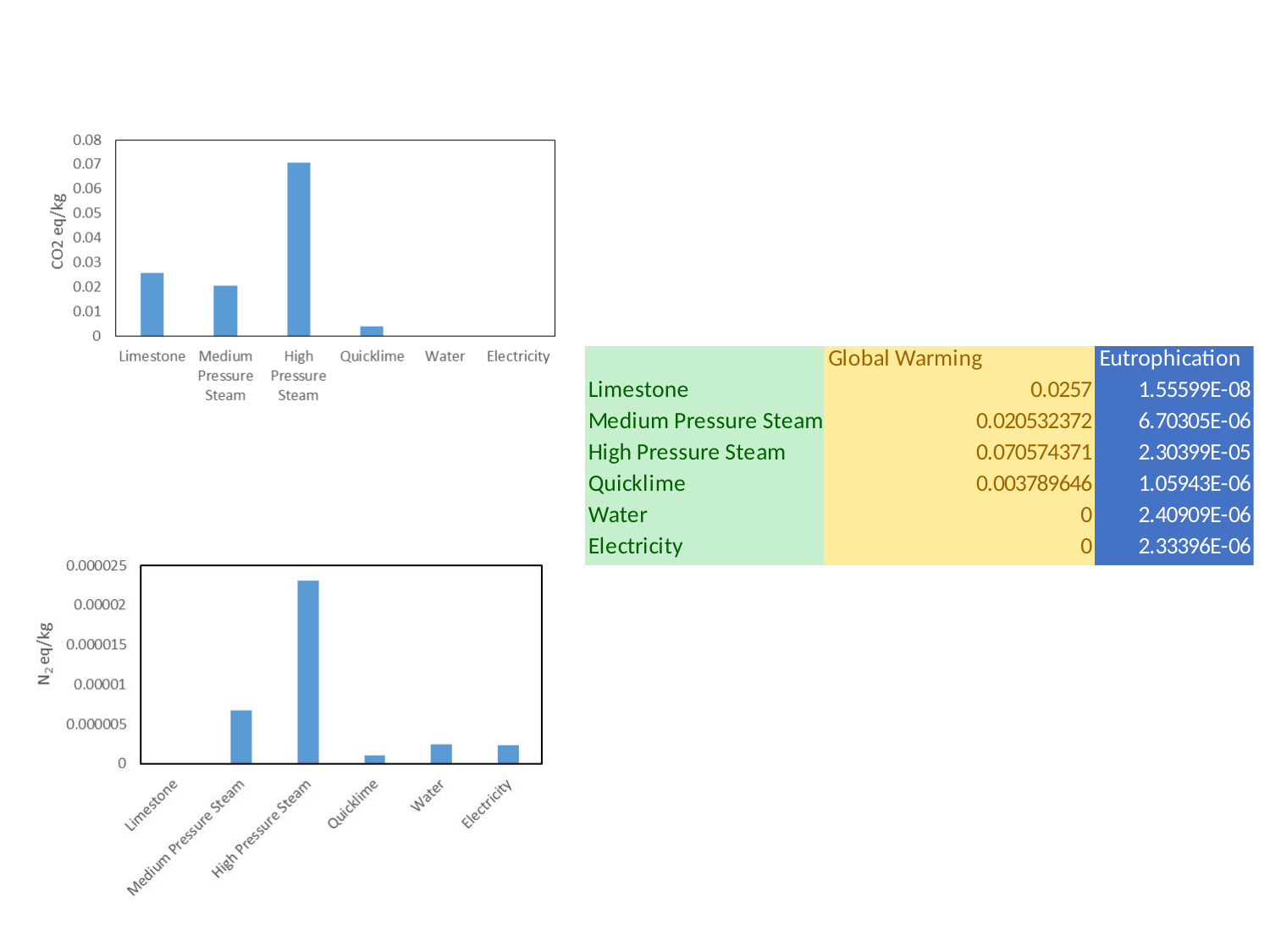

Supplement: Supplementary file 1 — 10.1186/s13068-016-0433-1 Reagent impacts. This is additional data from the study showing some of the eutrophication and global warming impacts of Reagents used in the study (i.e. limestone, medium/high pressure steam, quicklime, water). [file 13068_2016_433_MOESM1_ESM.pptx]
